# The role of landscape characteristics for forage maturation and nutritional benefits of migration in red deer

**DOI:** 10.1002/ece3.3006

**Published:** 2017-05-12

**Authors:** Atle Mysterud, Brit Karen Vike, Erling L. Meisingset, Inger Maren Rivrud

**Affiliations:** ^1^ Department of BiosciencesCentre for Ecological and Evolutionary SynthesisUniversity of OsloBlindernNorway; ^2^ Department of Forestry and Forestry ResourcesNorwegian Institute of Bioeconomy ResearchTingvollNorway

**Keywords:** elevation, movement ecology, normalized difference vegetation index, partial migration, seasonality, ungulates

## Abstract

Large herbivores gain nutritional benefits from following the sequential flush of newly emergent, high‐quality forage along environmental gradients in the landscape, termed green wave surfing. Which landscape characteristics underlie the environmental gradient causing the green wave and to what extent landscape characteristics alone explain individual variation in nutritional benefits remain unresolved questions. Here, we combine GPS data from 346 red deer (*Cervus elaphus*) from four partially migratory populations in Norway with the satellite‐derived normalized difference vegetation index (NDVI), an index of plant phenology. We quantify whether migratory deer had access to higher quality forage than resident deer, how landscape characteristics within summer home ranges affected nutritional benefits, and whether differences in landscape characteristics could explain differences in nutritional gain between migratory and resident deer. We found that migratory red deer gained access to higher quality forage than resident deer but that this difference persisted even after controlling for landscape characteristics within the summer home ranges. There was a positive effect of elevation on access to high‐quality forage, but only for migratory deer. We discuss how the landscape an ungulate inhabits may determine its responses to plant phenology and also highlight how individual behavior may influence nutritional gain beyond the effect of landscape.

## 1 Introduction

Migration between separate seasonal home ranges is a common phenomenon across animal taxa in many ecosystems all over the globe (Bauer & Hoye, 2014; Bolger, Newmark, Morrison, & Doak, 2008; Fryxell, Greever, & Sinclair, 1988). At northern latitudes with strong seasonality, large migratory herbivores move from winter to summer ranges when snow gradually melts in the spring and new vegetation of high nutritional quality emerges. Environmental gradients in the landscape cause a predictable sequence of a green flush of fresh, new growth, starting at low elevations (or latitudes), and moving toward higher elevations (or latitudes), a phenomenon referred to as the green wave (van der Graaf, Stahl, Klimkowska, Bakker, & Drent, 2006; Merkle et al., 2016). Early phenological growth stages of plants have higher nutritional quality due to high cell soluble content and low levels of defense compounds (Van Soest, 1994). The basis for the forage maturation hypothesis is that large migratory herbivores will preferentially follow phenological gradients or green waves in order to maximize access to the optimal combination of quality and quantity of forage (Albon & Langvatn, 1992; Fryxell & Sinclair, 1988; Hebblewhite, Merrill, & McDermid, 2008), ultimately resulting in increased body growth, reproductive rates, and survival rates (White, 1983).

There are now several studies providing empirical support of the forage maturation hypothesis, demonstrating that herbivores utilize spatial variation in the onset of plant growth to enhance the duration of access to newly emergent, high‐quality plants (Bischof et al., 2012; Hebblewhite et al., 2008; Merkle et al., 2016; Searle, Rice, Anderson, Bishop, & Hobbs, 2015). These studies have provided support for several predictions from the forage maturation hypothesis: (1) that migratory individuals gain access to a higher quality diet than resident individuals (Bischof et al., 2012; Hebblewhite et al., 2008), (2) that migratory individuals gain access to newly emergent plants by migrating between separate ranges compared to remaining in their winter ranges (Bischof et al., 2012), and (3) that herbivores actually follow the green wave (Merkle et al., 2016). However, there has been limited effort to relate the individual variation in access to newly emergent plants to the landscape characteristics that cause environmental gradients in the onset and development of plant growth, such as latitude, distance to coast, elevation, slope, and aspect. At high elevations and further inland, the snow cover remains for a longer time in the spring, and together with lower temperatures, this causes delayed forage development during the summer. The summer ranges of red deer (*Cervus elaphus*) inland and at higher elevations have higher forage quality in late summer (Albon & Langvatn, 1992). The forage quality at migration stop‐over sites was positively correlated with elevation for mule deer (*Odocoileus hemionus*) (Sawyer & Kauffman, 2011). We may also expect delayed phenology at sites with a more northerly aspect and along flat terrain; such sites allow snow to accumulate, delaying plant growth in the spring. It remains largely an open question which landscape variables other than elevation underlie the most beneficial phenological gradient for ungulates, yielding the highest access to high‐quality forage.

The aim of this study was to test how landscape characteristics, habitat type, and individual home range characteristics predict the access of 346 individual GPS‐marked red deer to newly emergent plants in four populations in the variable landscapes of Norway. The combination of GPS‐based telemetry and satellite images measuring the greenness of the vegetation, such as the normalized difference vegetation index (NDVI), now allow us to explore such relationships in detail (Bischof et al., 2012). We aim to test the following predictions from the forage maturation hypothesis: (P_1_) Migratory animals have access to higher quality forage (higher cumulative instantaneous rate of growth, CIRG) than resident animals. (P_2_) The variation in landscape characteristics in summer home ranges, such as elevation, distance to fjord, aspect, and slope, causes variation among individuals in terms of their access to high‐quality forage (CIRG). (P_3_) Variation in landscape characteristics in the summer home ranges explains the differences between migratory and resident deer in terms of access to high‐quality forage (CIRG). We further tested whether the effects of landscape characteristics affected resident and migratory deer in the same way (i.e., if there were interactions).

## 2 Material and Methods

### 2.1 Study area

The data were derived from four counties on the west coast of Norway: Hordaland, Sogn & Fjordane, Møre & Romsdal, and Sør‐Trøndelag, the core area for red deer in Norway in terms of historical distribution and population density. The study area has a diverse topography, from flat coastal areas to steep fjord landscapes and mountains. The temperature and snow depth increase from the coast to inland (Mysterud, Yoccoz, Stenseth, & Langvatn, 2000). The vegetation is in the boreonemoral zone for the most part, with a small proportion of Sør‐Trøndelag in the southern boreal zone and a small proportion of Hordaland in the nemoral zone (Abrahamsen et al., 1977). The natural forests are characterized by Scots pine (*Pinus sylvestris*) and deciduous trees such as birch (*Betula* spp.) and gray alder (*Alnus incana*). Planted Norway spruce (*Picea abies*) has a patchy distribution across the study area. Agricultural areas are mainly located on flatter ground near the coast or on valley floors. The cultivated land is mostly meadows and pastures for grass production (Lande, Loe, Skjærli, Meisingset, & Mysterud, 2014). Some grains (*Hordeum vulgare* and *Avena sativa*) are produced in the warmest and most fertile areas, particularly in Sør‐Trøndelag county.

### 2.2 Red deer data

We used GPS data from 346 collared red deer that were followed along the west coast of Norway in the period from 2004 to 2014. Subsets of the data have been used previously (Bischof et al., 2012; Mysterud et al., 2011; Rivrud et al., 2016). The procedure used to collar the red deer has been approved by the Norwegian Animal Research Authority, and the chemical immobilization and marking methods follow standard protocols (Sente et al., 2014). Adult deer (females ≥ 1.5 years; males ≥ 2.5 years) were marked with GPS collars (Tellus from Followit, Sweden, and GPS ProLite from Vectronic, Germany) and weighed to the nearest 0.5 kg. The collars were set to download a position every hour or every second hour. As some individuals were followed for more than 1 year, we only used data from the first recorded season per individual to avoid pseudoreplication. Locations recorded during the first 24 hr after marking were removed, and the raw data were screened for outliers (Bjørneraas, Van Moorter, Rolandsen, & Herfindal, 2010).

Space use tactics were determined using the Net‐Square Displacement (NSD) technique (Bunnefeld et al., 2011), but modified so that individual fit was assessed manually, as in our previous work (Bischof et al., 2012; Mysterud et al., 2011; Rivrud et al., 2016). We only included individuals classified as migrants (*n* = 190) or residents (*n* = 156).

### 2.3 Home range characteristics

As spring migrations in Norwegian red deer are rapid and closer to jumping than surfing in the wave use continuum (Bischof et al., 2012), we used the individual's summer home range as a basis for the demarcation of landscape characteristics. Ninety‐five percent utilization distribution home ranges were calculated using fixed‐kernel density estimation in the R package adehabitat (Calenge, 2006). The reference method was used to calculate the smoothing factor, *h*, for each individual. For resident red deer, GPS fixes from 1 April to 31 August were used to match with the growing season used in our analysis. Summer home ranges for migratory deer were calculated using GPS fixes from the time they reached the summer ranges until they departed back to winter ranges.

A range of landscape covariates was extracted from the individual home ranges by overlaying the home range polygons on the landscape maps, and the means of all pixel values within the home ranges were calculated. Slope (degrees; 0–90), aspect (continuous degrees; 0–360, where 0 is north and 180 is south), and elevation (m a.s.l.) were derived from a Digital Elevation Model (DEM). Aspect was further converted to northness (ranging from −1 to 1, where values close to −1 face south, and values close to 1 face north) by cosine transformation. Distance to outer coastline and distance to nearest fjord were measured in kilometers. In addition, the standard deviation of elevation was extracted for each home range as a measure of the variation in topography.

The proportions of different habitat types within the home ranges were derived from digital land resource maps at a scale of 1:50,000 (Loe, Bonenfant, Meisingset, & Mysterud, 2012). The eight original habitat types were simplified into four categories: pasture, forest, mountain (areas above the treeline), and all other habitat types (human settlement, marsh, water, glaciers, and areas not mapped). All landscape maps were rasterized with a resolution of 100 m. In the modeling, these variables were calculated as proportions within the seasonal home ranges of the individual deer.

One may argue that summer home range characteristics are not the only relevant scale when measuring nutritional gain and that the annual range is also important. We therefore also calculated the distance between summer and winter ranges and the difference in elevation between summer and winter ranges (Δelevation). The mean elevation of the winter and summer ranges was calculated based on the individual 95% fixed‐kernel home ranges for March (*n* = 290), representing winter, or April (*n* = 41) when data for March were not available, and for July (*n* = 334), representing summer, or June (*n* = 5) when July data were not available. The choice of these months as summer and winter ranges corresponded well with red deer migration dates, with a few exceptions for the winter range when individuals started spring migration toward the end of the chosen month (*n* = 8) or arrived in the summer range in the chosen month (*n* = 3). The centroids of individual 95% minimum convex polygons from the same months were used to calculate the distance between summer and winter ranges, as kernel home ranges often result in multiple polygons per individual, complicating centroid estimation.

### 2.4 Plant phenology from satellite NDVI

The NDVI is a measure of the reflected photosynthetic activity in a defined area (Pettorelli, Vik, et al., 2005) and is therefore commonly used as a proxy for forage quantity and quality for ungulates (Garroutte, Hansen, & Lawrence, 2016; Hamel, Garel, Festa‐Bianchet, Gaillard, & Côté, 2009). Data on the NDVI were extracted by downloading images covering Norway derived from the satellite MODIS TERRA (MOD13Q1) and available from the NASA Land Processes Distributed Active Archive Center website (https://lpdaac.usgs.gov/data_access/daac2disk ). The spatial resolution of these images is 250 m, and the temporal resolution is 16 days. For each 16‐day period, the images were merged and subsampled using the MODIS reprojection Tool v.4.1 (https://lpdaac.usgs.gov/tools/modis_reprojection_tool).

In accordance with our earlier study (Bischof et al., 2012), we extracted information about the instantaneous rate of green‐up (IRG), measuring the speed of the plant green‐up in spring. The IRG is defined as the first derivative of a double‐logistic function fitted to the annual time series of NDVI values scaled between 0 and 1 for a given pixel, that is, when the change in NDVI value peaks. This metric has been verified by independent testing (Merkle et al., 2016). A space–time–time matrix that relates red deer movement data to the changes in green‐up in space and time was constructed for each individual deer. For each individual red deer, we calculated the cumulative IRG (CIRG) over the entire growth season (1 April – 31 August) by summing the IRG for all pixels the animals used over the season at a given time. This represents the total instantaneous rate of green‐up experienced by an individual red deer throughout the growth season.

### 2.5 Statistical analyses

All statistical analyses were performed in R (R Development Core Team 2016). We used generalized linear mixed models in the library lme4 (Bates & Maechler, 2009). The response variable was the CIRG for the growth season. We used a random intercept for year to control for annual variations in the mean CIRG due to climatic variation and weather conditions. Sixteen observations (CIRG: *n* = 5; elevation: *n* = 1; Δelevation/distance summer‐winter: *n* = 10) were removed due to missing values in the covariates, leaving *n* = 330 individuals available for the analyses (182 migratory and 148 resident individuals). Marginal and conditional *R* ^2^ were calculated following Nakagawa and Schielzeth (2013).

For continuous variables, we log transformed or arcsine square root transformed (habitat type, measured as proportions) covariates where appropriate to increase fit and stabilize the variance. Note that although the use of arcsine square root transformation has been criticized (Warton & Hui, 2011), this is mainly in regard to its use for response variables and not for covariates as in our case. In addition, some covariates were also rescaled by centering on the mean and dividing by the standard deviation where needed, that is, standardizing, as covariates being on very different scales causes model convergence issues. All covariates were assessed for nonlinearity with the response variable using GAM plots in the library mgcv (Wood, 2006), and adequate parametrizations were chosen based on this. We also checked for correlations between all covariates, excluding the assumed least relevant one from the global model when *r *> |.6|. Categorical covariates included in the model were sex and space use tactic (migratory or resident).

We used model selection with Akaike Information Criterion (AIC) to find the most parsimonious model (Burnham & Anderson, 2002). We considered models within ΔAIC < 2 to be competitive (Burnham & Anderson, 2004). We tested all possible combinations of fixed effects in the library MuMIn (Barton, 2015), as even correlations <|0.6| can affect the dependency of the order in which covariates are entered, making different procedures of stepwise model selection unreliable. We also tested for interactions between landscape variables and space use tactics (i.e., resident/migratory). In the first model selection procedure, the variable “distance between summer and winter range” was not included. We therefore reran the model selection procedure excluding distance, as this allowed us to increase the sample size by 10 individuals (*n* = 340; 186 migratory and 154 resident individuals). An overview of the mean environmental variables for resident and migratory deer is given in Table 1. The difference in elevation between the summer and winter ranges (Δelevation) and variation in elevation within summer ranges were not included in the model selection process due to high correlation with the elevation of summer ranges for both migratory (*r *=* *.71 and .67, respectively) and resident (*r *=* *.67 and .91, respectively) individuals. Similarly, slope and distance to outer coastline were not included due to their high correlation with elevation (*r *=* *.66 and .72, respectively). We found similar results when conducting the model selection procedure in two steps, first with only landscape variables or only biological variables predicting the CIRG and then adding both sets of variables to the same model.

**Table 1:** Descriptive characteristics of the environmental variables within the summer home ranges (95% kernel) of resident (*n* = 154) and migratory (*n* = 186) red deer in Norway. Note that the cumulative instantaneous rate of growth (CIRG) is calculated over the entire growing season

	Resident		Migratory	
	Mean	*SD*	Mean	*SD*
Elevation (m a.s.l.)	221	171	403	196
Slope (°)	15.2	9.1	18.2	8.3
Aspect (°)	178	51	177	48
Distance to outer coast (km)	36.7	36.2	52.1	30.2
Distance to fjord (km)	2.7	4.3	10.8	15.1
Prop. pasture	0.13	0.09	0.07	0.08
Prop. forest	0.64	0.20	0.64	0.18
Prop. mountain	0.11	0.12	0.19	0.20
Prop. other	0.13	0.13	0.10	0.09
CIRG	31.2	14.8	36.8	14.3

## 3 Results

Several models were competitive (ΔAIC < 2) with the most parsimonious model (Table 2). The most parsimonious model (lowest AIC) was also the one with the fewest covariates and highest AICc weight and was chosen as the final model. The final model explaining differences in benefits related to forage maturation (CIRG) included the landscape variables elevation, proportion of forest and mountain, space use tactic (migration vs. residency), and the interaction between space use tactic and elevation (Table 2). The proportion of variance explained by the fixed effects alone was 0.151 (marginal *R* ^2^), and the proportion of variance explained by both fixed and random effects was 0.234 (conditional *R* ^2^).

**Table 2:** An overview of model selection results from generalized linear mixed‐effect models for the CIRG (cumulative instantaneous rate of green‐up) in the growing season, a measure of forage quality, within home ranges of red deer in Norway. All models that differ <2 ∆AIC from the best model are presented. x = term included in model. AICc = Akaike information criterion corrected for sample size. ∆AIC = difference in AIC value between the candidate model and the model with the lowest AIC value. Migr = migratory behavior. Other = other habitat types. The preferred model is marked in gray. Only variables and interactions included in the top models are presented in the table

Migr	Distance to fjord	Elevation	Northness	Sex	Forest	Mountain	Other	Pasture	Migr × elevation	Migr × forest	Migr × mountain	Migr × other	Degrees of freedom	Log likelihood	AICc	∆AIC	AICc weight
x		x			x	x			x				8	−1362.8	2742.1	0	0.013
x		x			x	x		x	x				9	−1361.9	2742.4	0.30	0.011
x		x			x	x	x		x				9	−1361.9	2742.4	0.35	0.011
x		x		x	x	x			x				9	−1362.3	2743.2	1.14	0.007
x		x		x	x	x		x	x				10	−1361.4	2743.5	1.42	0.006
x		x			x		x		x				8	−1363.6	2743.5	1.48	0.006
x		x			x	x			x	x			9	−1362.6	2743.7	1.60	0.006
x		x			x	x	x		x			x	10	−1361.5	2743.7	1.62	0.006
x		x			x	x		x	x	x			10	−1361.5	2743.7	1.65	0.006
x		x			x	x	x					x	9	−1362.6	2743.7	1.67	0.006
x		x	x		x	x			x				9	−1362.6	2743.7	1.68	0.006
x		x		x	x	x	x		x				10	−1361.6	2743.8	1.74	0.005
x	x	x			x	x			x				9	−1362.7	2743.8	1.78	0.005
x		x			x	x			x		x		9	−1362.7	2743.9	1.78	0.005
x		x			x	x		x	x		x		10	−1361.6	2743.9	1.80	0.005
x		x	x		x	x		x	x				10	−1361.7	2744.0	1.93	0.005

**Figure 1:**
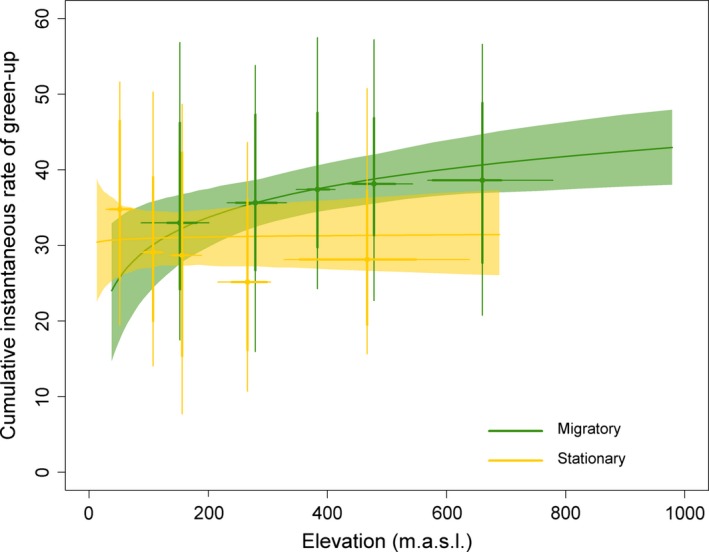
The cumulative instantaneous rate of green‐up (CIRG), a measure of forage quality obtained over the growing season, in relation to elevation (m a.s.l.) within home ranges of migratory (green; *n* = 186) and resident (yellow; *n* = 154) red deer in Norway. Lines are predicted means with 95% confidence intervals from the most parsimonious generalized linear mixed‐effect model. Points are averages of residuals for aggregated ranges of data: 20, 40, 60, 80, and 100th quantiles. Thin and thick lines indicate 80% and 50% of the data within these ranges, respectively

Migratory individuals had a higher CIRG than resident individuals (supporting P_1_), even after controlling for landscape characteristics, hence rejecting P_3_, which suggests that variation in the CIRG between migratory and resident deer is due to landscape characteristics only. Several of the landscape characteristics were significantly related to the CIRG (Table 3), providing overall support for P_2_, which suggests that variation in landscape characteristics in summer home ranges causes variation among individuals in their access to high‐quality forage (CIRG). Elevation was positively related to the CIRG for migratory individuals but not for resident individuals (Table 3; Figure 1). A 10% increase in the proportion of forest within the home range resulted in a mean CIRG that was 4.63 points lower (range 2.97 ‐ 9.49), while a 10% increase in the proportion of mountain resulted in a mean CIRG that was 1.89 points lower (range 1.21–3.88). All effect sizes are calculated on the original scale of the covariates (back‐transformed and unscaled).

**Table 3:** Parameter estimates of the resulting final generalized linear mixed effect model, explaining differences in the CIRG (cumulative instantaneous rate of green‐up) for the growing season, a measure of forage quality, within home ranges of red deer in Norway. *SE* = standard error. Migr = migratory behavior. Elevation was log transformed and rescaled by centering on the mean and dividing by the standard deviation, and the proportion of forest and mountain were arcsine square root transformed. Reference for migratory behavior is “migratory.” Standard deviation for random intercept “year” = 3.34. N_obs_ = 340

Fixed effects	Estimate	*SE*	*t*‐Value	*p*‐Value
Intercept	67.87	6.68	10.16	<.001
Migr: Resident	−5.16	1.69	−3.05	.002
Elevation	4.75	1.67	2.86	.005
Forest	−29.50	5.62	−5.25	<.001
Mountain	−12.06	4.78	−2.53	.012
Migr: Resident × elevation	−4.22	1.83	−2.30	.022

## 4 Discussion

We tested how much of the benefit migrants gain from forage maturation is due to the landscape characteristics of their summer home range. Migratory red deer gained access to higher quality forage than red deer that remained resident (P_1_), measured as the satellite‐derived NDVI cumulative instantaneous rate of green‐up, CIRG (Bischof et al., 2012; Merkle et al., 2016). It was clear that landscape characteristics such as elevation and habitat composition of the summer home range played a key role in causing individual variation in the CIRG (P_2_). However, the difference between migratory and resident red deer also remained after accounting for these landscape variables (contradicting P_3_).

### 4.1 Landscape does not fully explain why migratory deer benefit

It is well‐known that the plant phenological gradients utilized by migratory ungulates at northern latitudes are affected by several landscape characteristics, and our study provides further evidence for this. Several studies have shown that spring is delayed at higher elevations and that forage quality is therefore higher in late summer, such as for red deer ranges in Norway (Albon & Langvatn, 1992), elk ranges in the Canadian Rocky Mountains (Hebblewhite et al., 2008), and mule deer ranges in Wyoming, USA (Sawyer & Kauffman, 2011). In the Canadian Rocky Mountains, the delay was 50 days per 1,000 m elevation gain (Hebblewhite et al., 2008). Variation in topography leads to asynchronous plant phenology and in turn increases the body mass gain of deer following this phenology (Mysterud, Langvatn, Yoccoz, & Stenseth, 2001; Pettorelli, Mysterud, Yoccoz, Langvatn, & Stenseth, 2005; Searle et al., 2015). Any topographic feature creating spatial variation in forage emergence is likely to affect how high‐quality forage resources are available to deer during the growing season, and the specific landscape characteristics causing this likely differ among areas depending on topography.

Different landscape characteristics within summer home ranges are likely to be important factors explaining differences in the nutritional gain of resident and migratory deer. However, we found additional nutritional benefits of migration, as migrators still had higher estimates for CIRG compared to residents after landscape characteristics were controlled for. There can be several reasons for such a result. First, the scale of satellite images is likely too large to measure all variation in plant phenology. However, this cannot explain why migratory status remains significant after landscape characteristics have been controlled for. Second, our study is within the realm of optimal foraging theory using a single measure (the NDVI‐derived measure CIRG) for “nutritional benefit.” Such a framework has been criticized for not being nutritionally explicit and for being unidimensional (Raubenheimer, Simpson, & Mayntz, 2009). However, it is quite well‐known that during summer, protein is a key component of dietary choice and a key limiting nutritional factor, and the clear link between NDVI and fecal N suggest it is likely a good indicator of nutritional benefit (Hamel et al., 2009). In Yellowstone, the NDVI predicted both crude protein and in vitro dry matter digestibility of elk forage (Garroutte et al., 2016). Further, a polynomial model of the NDVI was better than a linear term (Garroutte et al., 2016), and this is good evidence that our approach using the derivative of the NDVI, IRG, is better than using the NDVI directly. Additionally, the fact that ungulates also select areas of high IRG provides further evidence that it is a relevant indicator of nutritional benefit (Merkle et al., 2016). Body condition is an integrator of nutritional intake and demands (Parker, Barboza, & Gillingham, 2009), and CIRG was related to body mass in male red deer (Bischof et al., 2012).

A key issue for further research is to understand better how the NDVI and the derived indexes IRG and CIRG relate to both plant quality and quantity when compared across habitats. The NDVI measures the amount of greenness in an image taken from above and thus also reflects the tree canopy if this is present. Our study areas have semi‐open forests, so the greening of the forest floor affects the NDVI. Importantly, the IRG (and CIRG) are measured as a *rate of change*, the derivative of the NDVI. In a coniferous forest, the canopy clearly affects the absolute value of the NDVI but unlikely affects the derivative of the NDVI in spring as much as the field layer vegetation. In a deciduous forest, the main spring flush arrives first in the field layer, and we regard it unlikely that the later greening of tree canopies will further increase the maximum rate of change of the NDVI. It would be preferable if it was possible to separate out only the forage species, but we are unlikely to arrive at that point in the near future given the scale of the sensors. The habitat composition within the home range nevertheless played a role in the CIRG, with a high proportion of forest areas decreasing in CIRG. Due to the unit‐sum‐constraint (Aebischer, Robertson, & Kenward, 1993), there will always be a level of correlation for a composition of habitat types within a home range, and it is therefore difficult to disentangle the detailed mechanism of how the CIRG is affected by habitat.

Further, we measured the benefit of migration over the entire growing season, 1 April–31 August, while we related landscape characteristics only to summer home range descriptors. The distance moved from winter to summer ranges positively affected forage quality in mule deer (Sawyer & Kauffman, 2011). Therefore, variability within the entire annual range may theoretically affect nutritional gain. Higher quality forage for migratory individuals was also found in a study on Sika deer (*Cervus nippon*) in Japan, in which only deer undergoing uphill migration experienced higher forage quality measured as fecal nitrogen during summer, while migrators at lower elevations did not (Sakuragi et al., 2003). However, in our case, the distance between the centers of the summer and winter ranges did not enter our most parsimonious model. Partial migration implies shared winter ranges to some extent and having a high‐elevation summer range should imply a broader gradient of the annual range. However, the difference in elevation between the summer and winter ranges (Δelevation) was correlated with the elevation of the summer range in both migratory and resident individuals. The fact that such a high correlation was also found in residents is interesting, as this implies that even many resident animals with overlapping seasonal ranges gain a considerable increase in elevation during summer. Surprisingly, the landscape effect interacted with space use tactic, as only migrants benefitted from having a summer range at a higher elevation (Figure 1). The elevation of the summer range was also correlated with the variation in elevation within the summer range, making it difficult to tease apart the relative roles of different detailed components of landscape characteristics.

From our study, we can conclude that a considerable part, but not all, of the benefit from migration is due to the landscape in which the animals live.

## Conflict of Interest

None declared.

## Authors' Contributions

AM designed the work. IMR performed the main statistical analysis, with smaller parts being performed by BV and AM. ELM organized data collection and database storage. AM drafted most of the first draft, with subsections being drafted by BV and IMR. All authors read and approved the final version.
